# Discovery of Novel Antibiotic Resistance Determinants in Forest and Grassland Soil Metagenomes

**DOI:** 10.3389/fmicb.2019.00460

**Published:** 2019-03-07

**Authors:** Inka Marie Willms, Aysha Kamran, Nils Frederik Aßmann, Denis Krone, Simon Henning Bolz, Fabian Fiedler, Heiko Nacke

**Affiliations:** ^1^Department of Genomic and Applied Microbiology and Göttingen Genomics Laboratory, Institute of Microbiology and Genetics, Georg-August University, Göttingen, Germany; ^2^Department of General Microbiology, Institute of Microbiology and Genetics, Georg-August University, Göttingen, Germany

**Keywords:** soil metagenome, functional metagenomics, antibiotic resistance, dihydropteroate synthase, tetracycline resistance, sulfonamide resistance

## Abstract

Soil represents a significant reservoir of antibiotic resistance genes (ARGs), which can potentially spread across distinct ecosystems and be acquired by pathogens threatening human as well as animal health. Currently, information on the identity and diversity of these genes, enabling anticipation of possible future resistance development in clinical environments and the livestock sector, is lacking. In this study, we applied functional metagenomics to discover novel sulfonamide as well as tetracycline resistance genes in soils derived from forest and grassland. Screening of soil metagenomic libraries revealed a total of eight so far unknown ARGs. The recovered genes originate from phylogenetically diverse soil bacteria (e.g., Actinobacteria, Chloroflexi, or Proteobacteria) and encode proteins with a minimum identity of 46% to other antibiotic resistance determinants. In particular forest soil ecosystems have so far been neglected in studies focusing on antibiotic resistance. Here, we detected for the first time non-mobile dihydropteroate synthase (DHPS) genes conferring resistance to sulfonamides in forest soil with no history of exposure to these synthetic drugs. In total, three sulfonamide resistant DHPSs, differing in taxonomic origin, were discovered in beech or pine forest soil. This indicates that sulfonamide resistance naturally occurs in forest-resident soil bacterial communities. Besides forest soil-derived sulfonamide resistance proteins, we also identified a DHPS affiliated to Chloroflexi in grassland soil. This enzyme and the other recovered DHPSs confer reduced susceptibility toward sulfamethazine, which is widely used in food animal production. With respect to tetracycline resistance, four efflux proteins affiliated to the major facilitator superfamily (MFS) were identified. Noteworthy, one of these proteins also conferred reduced susceptibility toward lincomycin.

## Introduction

Pathogenic bacteria resistant to multiple classes of antibiotics pose risks to public health and are considered as one of the major global challenges within the 21st century. Some of the antibiotic resistance genes (ARGs) carried by these bacteria have been traced to soil origins (Forsberg et al., 2012) and can potentially spread via e.g., groundwater or wildlife (Davies and Davies, 2010). Nevertheless, in contrast to clinical pathogens, bacterial communities inhabiting complex environments such as soil have been rarely considered within studies focusing on antibiotic resistance (Walsh, 2013b). To assess risks of environmental resistomes and develop strategies to tackle antibiotic resistance, an improved knowledge on the ecology of resistance determinants including their origins, diversity and underlying resistance mechanisms is urgently required (Allen et al., 2009; Wang et al., 2017).

Among Earth’s microbial habitats, soil harbors the highest diversity of prokaryotes including numerous multi-resistant bacteria (Delmont et al., 2011; Walsh and Duffy, 2013; Nesme and Simonet, 2015). The synthesis of antibiotics likely evolved in this habitat and promoted the development of different antimicrobial compound-specific resistance mechanisms (D’Costa et al., 2007; Walsh, 2013a). Previously unknown soil-derived ARGs were recovered from both, pristine and intensively managed sites, by function-based screening of metagenomic libraries (Allen et al., 2009; Perron et al., 2015; Lau et al., 2017). In contrast to sequence-based metagenomic library screening, this culture-independent approach is not based on conserved DNA regions and therefore allows the identification of entirely novel target genes (Nacke and Daniel, 2014; Cheng et al., 2017). For instance, a so far unknown peptide-associated macrolide resistance mechanism was uncovered by coupling function-based metagenomic library screening and high-resolution proteomics analysis (Lau et al., 2017). Besides dependence on conserved DNA regions, the fact that various resistance genes show high levels of similarity to genes encoding other cellular functions (Martínez, 2008; Perron et al., 2015) represents another limitation of sequence-based resistome analysis. An illustrative example are efflux pumps of the resistance-nodulation-division (RND) superfamily, which can confer antibiotic resistance, but can also transport proteins involved in cell division and nodulation, or both (Piddock, 2006; Perron et al., 2015).

In this study, we used function-based metagenomic library screening to identify so far unknown tetracycline and sulfonamide resistance genes in forest and grassland soil. Due to an excellent therapeutic index, few side effects, oral administration and low cost, tetracyclines belong to the most widely used classes of broad spectrum antibiotics in clinic (Thaker et al., 2010; Wang et al., 2017). After more than 60 years of excessive tetracycline usage, tetracycline resistance became one of the most abundant antibiotic resistances among clinical and commensal microbes (Wang et al., 2017). Another class of antibiotics, sulfonamides, is also commonly prescribed to people suffering from infections (Landers et al., 2012).

ARGs present in forests and grasslands, belonging to the most abundant terrestrial ecosystems worldwide, might become clinically relevant as they can potentially spread via lateral gene transfer. Here, we report the identification of four novel tetracycline and four previously unknown sulfonamide resistance genes derived from these ecosystems. Most of the proteins encoded by the novel ARGs showed low identity to already known antibiotic resistance determinants.

## Materials and Methods

### Site Description, Soil Sampling, and Metagenomic Library Construction

Soil samples were derived from forest and grassland sites of the German Biodiversity Exploratories Schorfheide-Chorin and Schwäbische Alb (Fischer et al., 2010). The land use intensity index (LUI) (Blüthgen et al., 2012) was calculated for all grassland sites. To account for interannual variation in management practices, the LUI was calculated from 2006 to 2008 (sampling year) (Table 1). LUI allows separate analysis of the intensity of grazing (calculated by considering numbers of grazing cattle, horses, or sheep, and duration of grazing with respect to each site), the mowing frequency, and the intensity of fertilization. Forest plots were dominated by European beech (*Fagus sylvatica*) or Scots pine (*Pinus sylvestris*) (Table 1).

**Table 1:** Characteristics of the study sites.

Site	Land use	Management	Treatment	Tree species	LUI (grazing, mowing, fertilization)
AEG2	Grassland	Meadow	Fertilized	NA	0.00, 2.07, 1.27
AEG3	Grassland	Meadow	Fertilized	NA	0.00, 2.76, 2.06
AEW9	Forest	Unmanaged forest	NA	Beech	NA
SEG6	Grassland	Mown pasture	Non-fertilized	NA	0.29, 1.38, 0.00
SEG8	Grassland	Pasture	Non-fertilized	NA	0.14, 0.69, 0.00
SEW2	Forest	Age class forest	NA	Pine	NA
SEW5	Forest	Age class forest	NA	Beech	NA

*The table lists the sites, land use, management type, treatment, dominant tree species, and LUI, land use index (calculated for 2006–2008) for grassland samples. AEG/AEW: sites located in the Biodiversity Exploratory Schwäbische Alb; SEG/SEW: sites located in the Biodiversity Exploratory Schorfheide-Chorin.*

The collection of the samples was performed previously as described by Nacke et al. (2011a). Descriptions of the soil characteristics are provided in Table 2. Total microbial community DNA was isolated from collected soil by employing the PowerSoil DNA isolation kit (MoBio Laboratories, Carlsbad, CA, United States) and metagenomic libraries, named AEG2, AEG3, and SEG8 were generated as described by Nacke et al. (2011b). The metagenomic libraries AEW9, SEG6, SEW2, and SEW5 were previously constructed (Nacke et al., 2011b). Names of constructed metagenomic libraries refer to the designation of the samples from which the libraries were derived.

**Table 2:** Basic properties of soil samples.

Sample	Soil type	pH	OC (g kg^-1^)	Total N (g kg^-1^)	C:N ratio
AEG2	Leptosol	6.9	72.3	7.2	10.1
AEG3	Leptosol	6.3	53.7	5.2	10.4
AEW9	Leptosol	6.4	60.0	4.5	13.4
SEG6	Histosol	5.2	284.1	23.9	11.9
SEG8	Gleysol	7.4	73.2	7.1	10.4
SEW2	Cambisol	3.5	17.0	1.0	16.7
SEW5	Cambisol	3.1	29.6	1.6	18.3

*AEG/AEW: soil samples derived from the Biodiversity Exploratory Schwäbische Alb; SEG/SEW: soil samples derived from the Biodiversity Exploratory Schorfheide-Chorin.*

### Antibiotic Resistance Screening and Sequence Analysis

The function-based screening was based on the ability of metagenomic library-bearing *Escherichia coli* clones to form colonies on LB agar medium containing 50 mg/L kanamycin, which selects for the screening vector pCR-XL-TOPO (Thermo Fisher Scientific, Braunschweig, Germany), and 5 mg/L tetracycline or 250 mg/L sulfamethoxazole. Colonies formed after incubation for 1–3 days at 37°C under aerobic conditions were picked for further study.

The recombinant plasmids derived from positive clones were sequenced by Microsynth Seqlab (Göttingen, Germany) using Sanger sequencing technology. All plasmid inserts were taxonomically classified using the software KAIJU (Menzel et al., 2016). An initial prediction of ORFs located on the inserts was performed by employing the ORF finder tool provided by the National Center for Biotechnology Information (NCBI) and the Artemis program (Rutherford et al., 2000; Wheeler et al., 2003). The results were verified and improved manually by e.g., GC frame plot and ribosome-binding site analysis. Subsequently, blast (Altschul et al., 1990) search against the NCBI non-redundant protein sequence database was performed. In addition, Resfams (Gibson et al., 2015), a recently generated database of protein families and associated profile hidden Markov models, representing all major ARG classes, was used for sequence comparisons. Blast searches against the ACLAME database (Leplae et al., 2010) version 0.4 and the Gypsy database (Llorens et al., 2011) release 2.0 were performed to identify mobile genetic elements. Moreover, the IS finder (database from 2018-09-11) (Siguier et al., 2006) was employed for identification of bacterial insertion sequences.

A neighbor-joining phylogenetic tree was constructed in MEGA (version 7.0) (Kumar et al., 2016) based on a ClustalW (Thompson et al., 1994) alignment of dihydropteroate synthase (DHPS) sequences. A total number of 1,000 bootstrap samplings were carried out to test the tree topology. Branches corresponding to partitions reproduced in less than 50% bootstrap replicates were collapsed. The evolutionary distances were computed using the number of differences method.

### Subcloning of ORFs Potentially Encoding Antibiotic Resistance

To verify if candidate ORFs encode antibiotic resistance, they were subcloned into vector pCR4-TOPO (Thermo Fisher Scientific) and subsequently introduced into *E. coli* TOP10. Two insert sequences (corresponding plasmids, pLAEG3_tet01 and pLSEG6_tet01) encoded proteins with similarity to members of the TetR family of regulators. In this case, the gene encoding the regulator as well as the potential ARG were subcloned together. In a first step, PCR was performed for amplification of candidate ORFs (including sequences potentially comprising promoters) from plasmid DNA. PCR primers are listed in Table 3. The PCR reaction mixture (50 μl) contained 10 μl 5-fold Phusion GC buffer, 200 μM of each of the four deoxynucleoside triphosphates, 5% DMSO, 0.2 μM of each primer, 1 U of Phusion HF DNA polymerase (Thermo Fisher Scientific), and approximately 20 ng of plasmid DNA. The following thermal cycling scheme was used: initial denaturation at 98°C for 1 min, 20 cycles of denaturation at 98°C for 1 min, annealing for 45 s (annealing temperatures, see Table 3), and extension at 72°C for 30 s per kb, followed by a final extension period at 72°C for 5 min. PCR products were purified using the QIAquick PCR purification kit (Qiagen, Hilden, Germany) according to the instructions of the manufacturer. Subsequently, a deoxyadenosine was added to the 3′ termini of the DNA as described by Nacke et al. (2011b) to facilitate cloning by the TA method. The DNA was then purified using the QIAquick PCR purification kit (Qiagen) and inserted into vector pCR4-TOPO (Thermo Fisher Scientific) as described by the manufacturer. Transformation of resulting vectors into *E. coli* TOP10 chemically competent cells was performed according to the protocol of the manufacturer.

**Table 3:** Primer sets designed in this study and corresponding templates.

Template	Oligonucleotide	Sequence (5′ to 3′)	Annealing temperature (°C)
pLAEG2_dhps01	AEG2_dhps01_for_150	GATACCCTAACGTACTACCGC	55
	AEG2_dhps01_rev	TCAGCGCGGATTCGTTC	55
pLAEW9_dhps01	AEW9_dhps01_for_150	CCTGATCGGTCAGGTCCTTA	55
	AEW9_dhps01_rev	TTACGCCGTTTGGCCC	55
pLSEW2_dhps01	SEW2_dhps01_for_150	CCGCCCGCCGTGTG	60
	SEW2_dhps01_rev	TTATGAAGCGGCGATAGCAGTAATAAC	60
pLSEW5_dhps01	SEW5_dhps01_for_104	GGTCATCGCGACAAAGGGTG	60
	SEW5_dhps01_rev	CTATACAGGCCGTCCAGCTGC	60
pLAEG3_tet01	AEG3_tet01b_for	CTATTGCTTGACGCGATCG	55
	AEG3_tet01a_rev	CTATTCCGCCGGCTCAG	55
pLSEG6_tet01	SEG6_tet01b_for	TTATCCTCGACGCGCCTTG	60
	SEG6_tet01a_rev	TCAGCCCGGAGCCAAGG	60
pLSEG8_tet01	SEG8_tet01_for_150	GGATTTGGAACAGACATATAGTG	55
	SEG8_tet01_rev	TTACCGGTTCCCCACTG	55
pLSEG8_tet02	SEG8_tet02_for_150	TTTAAGAGAATTTTCAGGATAAAG	50
	SEG8_tet02_rev	TTAACCATGCTTTGTCAG	50

### Antibiotic Susceptibility Analysis

Antibiotic susceptibility assays were conducted by using the 2-fold serial microtiter broth dilution method by considering the Clinical and Laboratory Standards Institute (CLSI) guidelines document M100-S24 (2014) and the MICs were recorded after 20 h of incubation at 37°C. The antibiotics cefotaxime, chloramphenicol, erythromycin, gentamicin, lincomycin, rifampicin, sulfadiazine, sulfamethoxazole, sulfamethazine, sulfisoxazole, tetracycline, and tylosin were considered. All assays were performed in duplicate. In addition, the susceptibility to different sulfonamides was further analyzed by spotting serial dilutions of cultures with starting OD_600_ of 0.5 onto Iso-Sensitest agar (Thermo Fisher Scientific) supplemented with sulfamethoxazole, sulfamethazine, sulfisoxazole or sulfadiazine. *E. coli* TOP10 carrying vector pCR4-TOPO (Thermo Fisher Scientific) was used as control.

### Accession Numbers

The insert sequences of the plasmids carried by metagenomic library clones showing decreased susceptibility to sulfamethoxazole or tetracycline have been submitted to GenBank under accession numbers MK159018 to MK159025.

## Results and Discussion

In order to discover so far unknown ARGs in environmental resistomes, soil metagenomic libraries were subjected to function-based screening. As sequence information is not required before screening, this is the only strategy that bears the potential to discover entirely novel ARGs (Simon and Daniel, 2009). In addition, it is selective for full-length genes and functional gene products. The soil used for construction of metagenomic libraries was derived from forest and grassland varying in land use history. Fertilized and non-fertilized grassland sites as well as pristine and age class forest sites, harboring different dominant tree species, were considered (Table 1). This enabled the identification of ARGs in soils from hardly as well as intensively managed ecosystems.

Metagenomic libraries contained approximately 39,800–559,000 clones (Table 4). The quality of the libraries was controlled by determining the average insert sizes and the percentage of insert-bearing *E. coli* clones. The average insert sizes of metagenomic DNA-containing plasmids ranged from 2.6 to 6.0 kb and the frequency of clones carrying plasmid inserts was at least 73% (Table 4).

**Table 4:** Characterization of soil metagenomic libraries and designation of plasmids harbored by positive clones.

Library	Number of clones	Average insert size (kb)	Insert frequency (%)	Estimated library size (Gb)	Plasmids of positive clones
AEG2	115965	3.6	73	0.30	pLAEG2_dhps01
AEG3	40095	5.8	85	0.20	pLAEG3_tet01
AEW9^∗^	100950	2.6	89	0.23	pLAEW9_dhps01
SEG6^∗^	39825	6.0	91	0.22	pLSEG6_tet01
SEG8	559000	4.8	86	2.30	pLSEG8_tet01-02
SEW2^∗^	135240	5.7	95	0.73	pLSEW2_dhps01
SEW5^∗^	166040	4.0	95	0.63	pLSEW5_dhps01

*AEG/AEW: metagenomic libraries derived from the Biodiversity Exploratory Schwäbische Alb; SEG/SEW: metagenomic libraries derived from the Biodiversity Exploratory Schorfheide-Chorin. ^∗^Previously generated libraries. Names of constructed metagenomic libraries refer to the designation of the samples from which the libraries were derived (see Table 2).*

### Novel ARGs Derived From Phylogenetically Divergent Soil Bacteria

The soil-derived metagenomic libraries were screened for resistance against tetracycline and sulfamethoxazole using selective agar medium. We recovered eight positive *E. coli* clones, harboring plasmids listed in Table 4, from functional screens. The entire inserts of these plasmids were sequenced and taxonomically classified, which revealed in all cases a bacterial origin (Supplementary Table S1). Some of the insert sequences are affiliated to Gram-negative bacterial phyla including Bacteroidetes and Proteobacteria whereas others belong to Actinobacteria (Supplementary Table S1). Noteworthy, one of the insert sequences was affiliated to the poorly characterized candidate phylum Zixibacteria.

Forsberg et al. (2014) reported that bacterial phyla, which were abundant in soil samples as determined by 16S rRNA gene sequencing, were also well-represented among taxa inferred from antibiotic resistance-conferring metagenomic library inserts derived from the same samples. Previously, we detected Proteobacteria, Actinobacteria, Bacteroidetes, and Chloroflexi among the dominant phyla in soils of our study sites via pyrosequencing of 16S rRNA genes (Kaiser et al., 2016). These phyla were also covered by the antibiotic resistance-conferring inserts described in this study (see Supplementary Table S1). Despite their high-GC content and predicted transcriptional incompatibilities with *E. coli*, also Actinobacteria were represented with respect to inserts of positive clones reported here and by Forsberg et al. (2014). The taxonomic origins of our resistance-conferring inserts show that the metagenomic library host *E. coli* allows identification of ARGs carried by phylogenetically divergent soil bacteria.

### Forest Soil Not Exposed to Synthetic Drugs Harbors Sulfonamide-Resistant DHPSs

**FIGURE 1:**
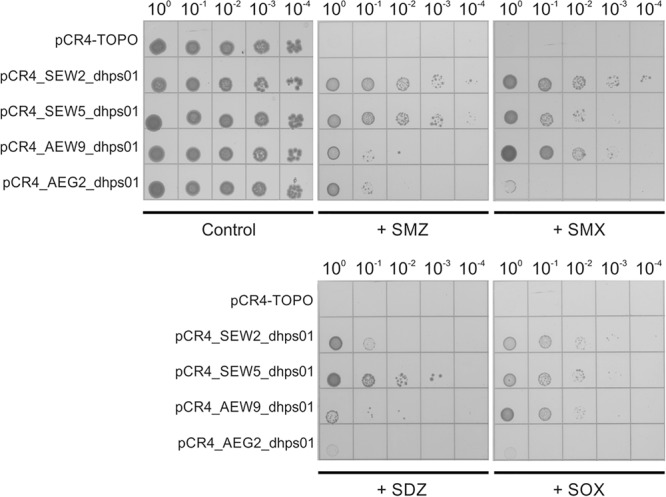
Resistance against sulfonamide antibiotics mediated by *SEW2_dhps01, SEW5_dhps01, AEW9_dhps01*, and *AEG2_dhps01*. Five microliters of serially diluted *E. coli* TOP10 cultures with starting OD_600_ of 0.5 were spotted onto Iso-Sensitest agar plates supplemented with 1000 mg/L sulfamethazine (+ SMZ), 250 mg/L sulfamethoxazole (+ SMX), 250 mg/L sulfadiazine (+ SDZ) or 500 mg/L sulfisoxazole (+ SOX). Iso-Sensitest agar plates with no sulfonamide added (control) were also included. *E. coli* TOP10 cultures carrying the cloning vector pCR4-TOPO, pCR4_SEW2_dhps01, pCR4_SEW5_dhps01, pCR4_AEW9_dhps01 or pCR4_AEG2_dhps01 were considered.

Sulfonamides are synthetic antimicrobial compounds targeting the folic acid pathway enzyme DHPS. Although all forest sites analyzed in this study exhibit no history of exposure to these synthetic compounds, three genes, *AEW9_dhps01, SEW2_dhps01*, and *SEW5_dhps01*, conferring sulfonamide resistance, were recovered from beech or pine forest soil (Tables 1, 5 and Figure 1). Furthermore, with respect to both forest sites (SEW2 and SEW5) located in the Schorfheide-Chorin exploratory (Northeastern Germany), as well as the forest site (AEW9) located in the Schwäbische Alb exploratory (Southwestern Germany), to our knowledge soils were not exposed to chemicals that resemble sulfonamides in their molecular structure. Especially, in case of the site AEW9 it is unlikely that such chemicals were spread, as this site belongs to an unmanaged beech forest. Resistance to sulfonamides is commonly mediated by the mobile DHPS-encoding genes *sul1, sul2*, or *sul3* (Sköld, 2000; Perreten and Boerlin, 2003), which have been detected in various environments such as shrimp ponds, swine farm wastewater and manured soil (Phuong Hoa et al., 2008; Wang et al., 2014), but also in clinical isolates (Grape et al., 2003). To our knowledge, we report here for the first time the presence of functional non-mobile sulfonamide-resistant DHPSs in forest soil ecosystems. The deduced gene products of *AEW9_dhps01, SEW2_dhps01*, and *SEW5_dhps01* showed only 46 to 58% amino acid sequence identities to the closest known DHPSs over the full length proteins (Table 5). Furthermore, *AEW9_dhps01* harbors the alternative start codon GTG (all other detected *dhps* genes harbored the start codon ATG).

**Table 5:** Proteins encoded by genes associated with antibiotic resistance and their observed sequence identities.

Plasmid	Gene	No. of encoded amino acids	Closest similar non-hypothetical protein, accession no. (no. of encoded amino acids), organism	*E*-value	Percent identity to the closest similar protein (Blast)	Percent identity to the closest similar protein (ClustalW alignment)
pLAEG2_dhps01	*AEG2_dhps01*	286	Dihydropteroate synthase, WP_116719066 (292), Anaerolineaeles bacterium	3e-151	213/282 (76%)	74%
pLAEW9_dhps01	*AEW9_dhps01*	273	Sulfonamide-resistant dihydropteroate synthase Sul3, WP_106052391 (263), Victivallales	1e-73	123/264 (47%)	46%
pLSEW2_dhps01	*SEW2_dhps01*	269	Dihydropteroate synthase, OGQ04760 (263), Deltaproteobacteria bacterium	3e-77	140/270 (52%)	52%
pLSEW5_dhps01	*SEW5_dhps01*	271	Dihydropteroate synthase, OJU07522 (270), Alphaproteobacteria bacterium 64-11	3e-99	159/259 (61%)	58%
pLAEG3_tet01	*AEG3_tet01a*	403	MFS transporter, AIA16595 (403), uncultured bacterium	0.0	398/403 (99%)	98%
	*AEG3_tet01b*	230	Bacterial regulatory protein of tetR family, AIA16695 (190), uncultured bacterium	2e-127	179/190 (94%)	94%
pLSEG6_tet01	*SEG6_tet01a*	408	MFS transporter, WP_078811785 (418), *Prosthecobacter debontii*	2e-128	200/383 (52%)	49%
	*SEG6_tet01b*	197	TetR family transcriptional regulator, PZN78209 (205), Proteobacteria bacterium	4e-63	109/194 (56%)	54%
pLSEG8_tet01	*SEG8_tet01*	432	MFS transporter, WP075350247 (408), *Algoriphagus marinus*	3e-174	250/402 (62%)	61%
pLSEG8_tet02	*SEG8_tet02*	405	Tetracycline resistance MFS efflux pump, AIA16766 (418), uncultured bacterium	0.0	272/402 (68%)	67%

**FIGURE 2:**
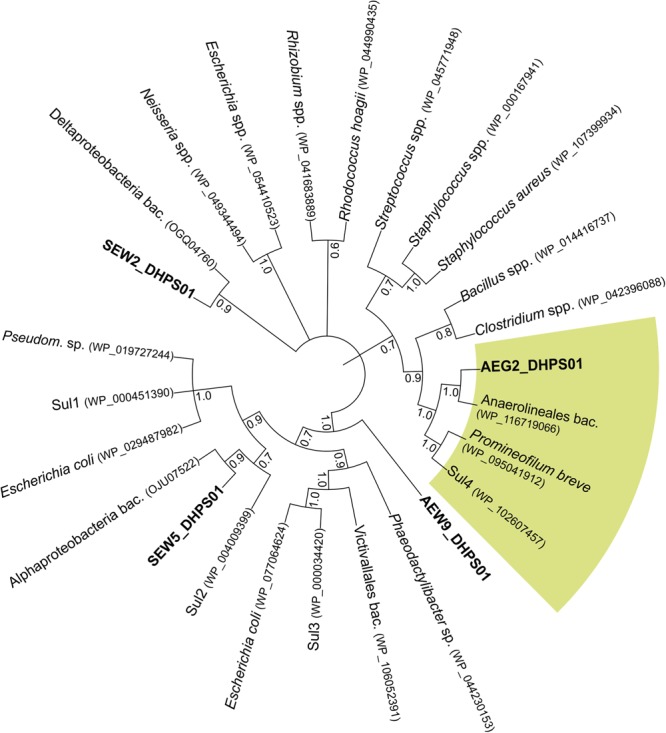
Neighbor-joining phylogenetic tree based on amino acid sequences of SEW2_DHPS01, SEW5_DHPS01, AEW9_DHPS01, AEG2_DHPS01 and other bacterial DHPSs. Besides DHPSs identified in this study, their closest related reference database entries, the mobile sulfonamide-resistant Sul1, Sul2, Sul3 and Sul4, and further DHPSs were considered. Bootstrap values based on 1,000 replicates are shown at the branching points. Branches are annotated with the identified taxon name and accession number in parentheses. The green segment indicates DHPSs belonging to Chloroflexi. *Pseudom*., *Pseudomonas*; bac., bacterium.

**FIGURE 3:**
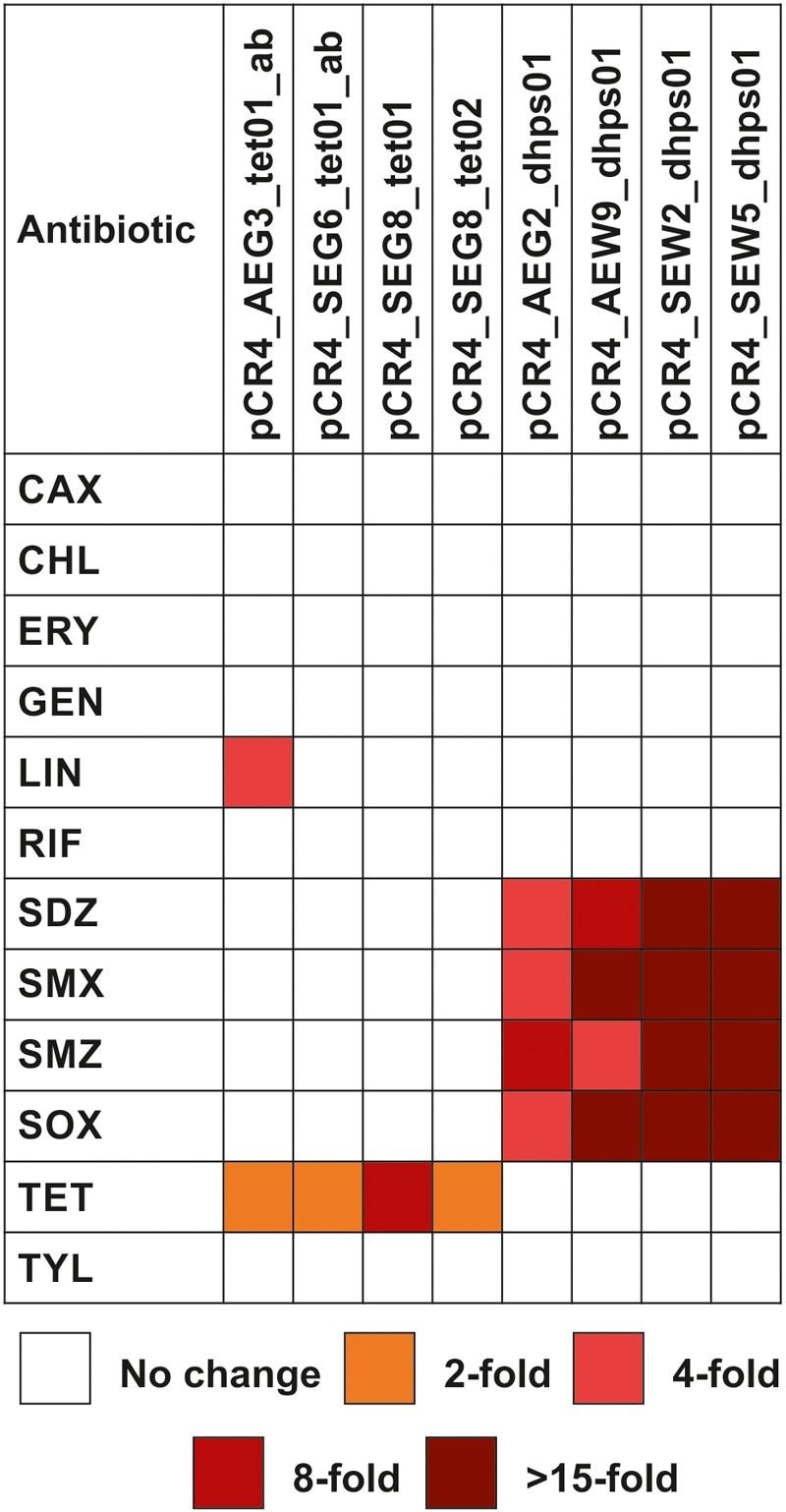
Antibiotic susceptibility profiles of *E. coli* TOP10 carrying soil-derived genes involved in antibiotic resistance. The genes were subcloned into plasmid vector pCR4-TOPO. MICs of antibiotics were determined using the broth microdilution method and are presented as fold increase relative to those for *E. coli* TOP10 carrying the cloning vector pCR4-TOPO. CAX, cefotaxime; CHL, chloramphenicol; ERY, erythromycin; GEN, gentamicin; LIN, lincomycin; RIF, rifampicin; SDZ, sulfadiazine; SMX, sulfamethoxazole; SMZ, sulfamethazine; SOX, sulfisoxazole; TET, tetracycline; TYL, tylosin.

Phylogenetic analysis revealed that SEW2_DHPS01 exhibits homology with a putative DHPS affiliated to Deltaproteobacteria (Figure 2). Nevertheless, it has so far not been analyzed if this putative enzyme represents a functional DHPS, which can confer resistance to sulfonamides. In contrast to SEW2_DHPS01, AEW9_DHPS01 showed low identity (46%) to a DHPS with confirmed sulfonamide resistance, but branched separately from this enzyme affiliated to the Lentisphaerae (family Victivallales), in a phylogenetic tree (Figure 2). The remaining sulfonamide resistance-conferring enzyme identified in forest soil, SEW5_DHPS01, was most similar (58% identity) to a DHPS from Alphaproteobacteria. Strikingly, no mobile genetic elements were predicted with respect to the inserts comprising *AEW9_dhps01, SEW2_dhps01*, and *SEW5_dhps01*. This indicates that different bacterial phyla colonizing forest soil ecosystems harbor DHPSs, which are naturally insensitive to the inhibitory effects of sulfonamides. Furthermore, our results show that forest soil-derived DHPSs can provide high-level resistance in *E. coli* TOP10 (Figure 1, 3) and therefore potentially also in clinically relevant Enterobacteriaceae. As sulfonamides are used to treat gastrointestinal or urinary infections in human and belong to the most commonly sold and administered veterinary antibiotics (De Briyne et al., 2014; Santman-Berends et al., 2014), mobilization and spread of so far unknown genes conferring resistance to these synthetic compounds would have severe consequences, especially for the animal sector. In particular, SEW2_DHPS01 and SEW5_DHPS01 exhibited high-level resistance toward sulfamethazine (Figure 1), which is widely used in food animal production (Lau et al., 2017).

### Discovery of a Grassland Soil-Derived DHPS Affiliated to Chloroflexi

Recently, a fourth mobile sulfonamide resistance gene (*sul4*), encoding a DHPS phylogenetically related to representatives of the phylum Chloroflexi, has been discovered in polluted Indian river sediment (Razavi et al., 2017). This gene is flanked by an IS*CR* element, which is known to be involved in horizontal gene transfer (Razavi et al., 2017). In this study, we identified an enzyme (AEG2_DHPS01) with reduced susceptibility toward sulfonamides (Figure 1 and Table 6), showing similarity to DHPSs from Chloroflexi, in a fertilized grassland soil. AEG2_DHPS01 shares 76% sequence identity with a DHPS from a member of the Anaerolineae (Table 5) and clusters with different Chloroflexi DHPSs including Sul4 in a phylogenetic tree (Figure 2).

**Table 6:** Antibiotic susceptibility of plasmid-carrying *E. coli* clones.

Plasmid	Minimal inhibitory concentration (μg/ml)											
	CAX	CHL	ERY	GEN	LIN	RIF	SDZ	SMX	SMZ	SOX	TET	TYL
Cloning vector	<0.125	2	1024	4	512	8	15.625	7.8125	62.5	15.625	1	512
pCR4_AEG2_dhps01	<0.125	2	1024	4	512	8	**62.5**	**31.25**	**500**	**62.5**	1	512
pCR4_AEW9_dhps01	<0.125	2	1024	4	512	8	**125**	**500**	**250**	**1000**	1	512
pCR4_SEW2_dhps01	<0.125	2	1024	4	512	8	**500**	**500**	**>1000**	**1000**	1	512
pCR4_SEW5_dhps01	<0.125	2	1024	4	512	8	**>1000**	**500**	**>1000**	**1000**	1	512
pCR4_AEG3_tet01ab	<0.125	2	1024	4	**2048**	8	15.625	7.8125	62.5	15.625	**2**	512
pCR4_SEG6_tet01ab	<0.125	2	1024	4	512	8	15.625	7.8125	62.5	15.625	**2**	512
pCR4_SEG8_tet01	<0.125	2	1024	4	512	8	15.625	7.8125	62.5	15.625	**8**	512
pCR4_SEG8_tet02	<0.125	2	1024	4	512	8	15.625	7.8125	62.5	15.625	**2**	512

*CAX, cefotaxime; CHL, chloramphenicol; ERY, erythromycin; GEN, gentamicin; LIN, lincomycin; RIF, rifampicin; SDZ, sulfadiazine; SMX, sulfamethoxazole; SMZ, sulfamethazine; SOX, sulfisoxazole; TET, tetracycline; TYL, tylosin. Bold values indicate an increase in minimal inhibitory concentration compared to the control strain carrying the cloning vector pCR4-TOPO.*

As *sul4* is flanked by a partial *folK* ORF, it might have been decontextualized from a set of chromosomal genes involved in folate synthesis (Razavi et al., 2017). Nevertheless, Razavi et al. (2017) pointed out that further investigations on Chloroflexi could provide additional hints about the original host of *sul4* and how it has been decontextualized. With respect to the insert carrying *AEG2_dhps01*, no genes potentially involved in folate synthesis were identified. Instead, *AEG2_dhps01* is flanked by an ORF encoding a putative gene product with low similarity (23% identity) to a primosomal protein N′ (replication factor Y) – superfamily 2 helicase from an *Anaerolineae bacterium* (Supplementary Table S2). It is possible that this gene product can contribute to horizontal gene transfer between Chloroflexi and other bacterial taxa as helicases play a major role in replication, recombination, and repair of nucleic acid substrates (Flechsig et al., 2011; Byrd and Raney, 2012). Besides the potential helicase gene, *AEG2_dhps01* is flanked by an ORF encoding a gene product with similarity to a hypothetical protein of an Anaerolineales bacterium.

Taxonomic analysis of the complete insert carrying *AEG2_dhps01* confirmed that its original host belongs to the Chloroflexi (Supplementary Table S1). Thus, besides Sul4, AEG2_DHPS01 represents the so far only identified DHPS showing reduced susceptibility toward sulfonamides (Table 6), which is affiliated to the Chloroflexi. In order to analyze if sulfonamide resistance is a common characteristic of Chloroflexi, isolates belonging to this phylum should be analyzed with respect to susceptibility toward synthetic drugs in future surveys. Apart from sulfonamides, no decreased susceptibility toward other tested antibiotics was detected with respect to *E. coli* TOP10 carrying the subcloned *dhps* genes (Figure 3 and Table 6).

### An Efflux Protein Conferring Reduced Tetracycline and Lincomycin Susceptibility

We identified four plasmids, pLAEG3_tet01, pLSEG6_tet01, pLSEG8_tet01, and pLSEG8_tet02, conferring efflux-mediated tetracycline resistance. All of these plasmids encode gene products with similarity to major facilitator superfamily (MFS) efflux proteins (Table 5). MFS efflux systems are widely distributed in both Gram-positive and Gram-negative bacteria (Sun et al., 2014). Accordingly, Wang et al. (2017) reported that 21 out of 24 tetracycline resistance genes, identified by functional metagenomics in Chinese soils, were affiliated to the MFS. The proteins encoded by these 21 genes showed identities ≥78% to the closest related reference database entries (Wang et al., 2017). In contrast, three out of four MFS representatives identified in this study shared ≤67% identity with their closest related proteins (Table 5). Besides an MFS representative, two insert sequences (corresponding plasmids, pLAEG3_tet01 and pLSEG6_tet01) encoded proteins with similarity to members of the TetR family of regulators (Table 5). These regulators are associated with antibiotic resistance and are known to control expression of MFS members (Cuthbertson and Nodwell, 2013). Noteworthy, the insert of plasmid pLtetSEG8_02 encodes a protein with similarity to an endonuclease (Supplementary Table S2), which might contribute to horizontal gene transfer.

McGarvey et al. (2012) identified a tetracycline-resistant metagenomic library clone, harboring a MFS representative, with reduced susceptibility toward rifampicin. Here, no resistance toward rifampicin was detected with respect to recombinant MFS producing *E. coli* clones (Figure 3). Nevertheless, the tetracycline-resistant clone carrying plasmid pCR4_AEG3_tet01ab showed reduced susceptibility toward lincomycin (Figure 3 and Table 6). The gene product AEG3_Tet01a encoded by this plasmid shows 98% identity to a soil-derived MFS from an uncultured bacterium (Table 5), which confers resistance to chloramphenicol. So far, it has not been analyzed if this chloramphenicol resistance mediating MFS identified by Forsberg et al. (2014) also encodes lincomycin resistance.

## Conclusion

Our findings highlight the vast potential of functional metagenomics for the discovery of so far unknown antibiotic resistance determinants in environmental resistomes. We recovered several soil-derived target genes and proteins with low similarity to reference database entries from hardly as well as intensively managed forest and grassland, indicating that the resistance reservoir of the uncultured microbial majority is far from being extensively explored. As we detected here for the first time non-mobile DHPSs conferring resistance to sulfonamides in forest soil with no history of exposure to these synthetic drugs, it is possible that this characteristic naturally occurs in complex bacterial communities. Most of the detected antibiotic resistance determinants were not flanked by potential mobile genetic elements. Nevertheless, the recent finding of a fourth mobile sulfonamide resistance gene indicates ongoing forces that introduce, mobilize and maintain antibiotic resistance determinants in bacterial communities (Razavi et al., 2017). Considering, that several ARGs reported here conferred high-level resistance to non-pathogenic *E. coli*, it can be assumed that this could also be the case with respect to clinically relevant Enterobacteriaceae. In order to predict the emergence of antibiotic resistance, an extensive knowledge on environmental resistomes will be required, which might also direct the design of novel antibiotics that are less susceptible to resistance.

## Data Availability

The datasets generated for this study can be found in GenBank, MK159018 to MK159025.

## Author Contributions

HN designed the study. IW, AK, NA, DK, SB, FF, and HN carried out field and laboratory work. IW and HN prepared and analyzed the data. All authors interpreted the results and wrote the manuscript.

## Conflict of Interest Statement

The authors declare that the research was conducted in the absence of any commercial or financial relationships that could be construed as a potential conflict of interest.
