# Supplementary material for: Discovery of Novel Antibiotic Resistance Determinants in Forest and Grassland Soil Metagenomes
Source: Front Microbiol. 2019 Mar 7;10:460. doi: 10.3389/fmicb.2019.00460 (PMC6416219; doi:10.3389/fmicb.2019.00460)
Supplement: Supplementary file 1 [file Table_1.DOCX]

Table S1. Taxonomic classification of plasmid inserts from positive clones.

| Plasmid | Taxonomic classification of insert |
| --- | --- |
| pLAEG2_dhps01 | Cellular organisms; Bacteria; Terrabacteria group; Chloroflexi; unclassified Chloroflexi; unclassified Chloroflexi (miscellaneous); Chloroflexi bacterium OLB14 |
| pLAEW9_dhps01 | Cellular organisms; Bacteria; Terrabacteria group; Actinobacteria; Actinobacteria; Micrococcales; Microbacteriaceae; *Microbacterium*; *Microbacterium* sp. |
| pLSEW2_dhps01 | Cellular organisms; Bacteria; unclassified Bacteria; Bacteria candidate phyla; candidate division Zixibacteria; candidate division Zixibacteria bacterium SM23_81 |
| pLSEW5_dhps01 | Cellular organisms; Bacteria; Proteobacteria; Alphaproteobacteria; Rhizobiales; Rhodobiaceae; *Parvibaculum*; *Parvibaculum lavamentivorans* |
| pLAEG3_tet01 | Cellular organisms; Bacteria; environmental samples; uncultured bacterium |
| pLSEG6_tet01 | Cellular organisms; Bacteria; Terrabacteria group; Actinobacteria; Actinobacteria; Corynebacteriales; Mycobacteriaceae; *Mycobacterium*; *Mycobacterium rhodesiae* |
| pLSEG8_tet01 | Cellular organisms; Bacteria |
| pLSEG8_tet02 | Cellular organisms; Bacteria; FCB group; Bacteroidetes/Chlorobi group; Bacteroidetes; Chitinophagia; Chitinophagales |
