# Supplementary material for: Discovery of Novel Antibiotic Resistance Determinants in Forest and Grassland Soil Metagenomes
Source: Front Microbiol. 2019 Mar 7;10:460. doi: 10.3389/fmicb.2019.00460 (PMC6416219; doi:10.3389/fmicb.2019.00460)
Supplement: Supplementary file 2 [file Table_2.DOCX]

Table S2. Open reading frames potentially involved in lateral gene transfer identified on plasmids and description of corresponding gene products and their observed sequence identities.

| Plasmid | ORF# | No. of encoded amino acids | Closest similar protein potentially involved in lateral gene transfer, accession no. (no. of encoded amino acids), organism | E value | Percent identity to the closest similar bacterial protein |
| --- | --- | --- | --- | --- | --- |
| pLAEG2_dhps01 | 18 | 445 | Primosomal protein N’ - superfamily II helicase, RCK75038 (471), Anaerolineae bacterium | 1e-40 | 118/429 (28%) |
| pLSEG8_tet02 | 40 | 123 | Endonuclease domain-containing protein, WP_068706482 (129), Paludibacter jiangxiensis | 4e-35 | 57/115 (50%) |
